# Population-level gender-based analysis of the educational journeys of students with autism spectrum disorder in British Columbia, Canada

**DOI:** 10.1177/13623613251345532

**Published:** 2025-06-21

**Authors:** Jennifer Baumbusch, Jennifer EV Lloyd, Vanessa C Fong

**Affiliations:** The University of British Columbia, Canada

**Keywords:** autism spectrum disorder, British Columbia, Canada, educational outcomes, gender differences, high school completion, population data

## Abstract

Research examining the impact of gender on educational outcomes in autistic students has been sparse. To address this gap, this study investigated the educational journeys of students with autism spectrum disorder in British Columbia, Canada. We examined (1) the time it takes for students to receive an initial autism spectrum disorder designation; and the frequency and percentage of students who (2) stay longitudinally in the Kindergarten-to-Grade 12 school system; (3) complete high school and the credential earned; and (4) proceed to public post-secondary education within British Columbia. We conducted secondary analyses of administrative data. The autism spectrum disorder student population was divided into eight longitudinal cohorts with a combined final sample size of 4282 students with autism spectrum disorder: 738 female (17.2%) and 3544 (82.8%) male. Descriptive analyses indicated statistically significant gender differences in students’ time to initial autism spectrum disorder designation, rates of high school completion and the specific high school credential earned. No gender differences were found in post-secondary transition rates. During their formative education years, gender differences, particularly the delay in autism spectrum disorder diagnosis among girls, may have implications with respect to educational outcomes. Results emphasize the need to provide educators with greater information about recognizing gender differences in autism spectrum disorder.

**Lay Abstract:**

a. What is already known about the topic?

Over the past several years, there is growing acknowledgement of gender inequities among people with autism spectrum disorder. The inequity is evidenced, in part, by gender differences in diagnosis. Although the gender gap is narrowing, until recently the diagnostic criteria for autism spectrum disorder has largely favoured and is more sensitive to detecting autism spectrum disorder in boys.

b. What does this paper add?

Research examining the impact of gender on educational outcomes in autistic students has been sparse. To address this gap in the literature, the current study investigated the educational journeys of students with autism spectrum disorder in British Columbia, Canada.

We found statistically significant gender differences in students’ time to initial autism spectrum disorder designation, rates of high school completion and the specific high school credential earned. There were, however, no significant differences in whether or not students stayed longitudinally in the K-12 school system over time, whether students transitioned into post-secondary or not (non-developmental or developmental), nor in students’ transition times into the respective post-secondary education programmes.

This study highlights the value of longitudinal, population-based and student-level data in conducting gender-based analyses in autism spectrum disorder research.

c. Implications for practice, research or policy

Understanding how gender impacts the academic trajectories of students with autism spectrum disorder over time can inform the development of tailored interventions and services which address their unique needs. Ultimately, this research is needed to promote more equitable educational experiences and outcomes.

There is growing acknowledgement of gender inequities among people with autism spectrum disorder (hereafter, ASD) (Loomes et al., 2017; Tsirgiotis et al., 2022). The inequity is evidenced, in part, by gender differences in diagnosis. Although the gender gap is narrowing, until recently, diagnostic criteria were more sensitive to detecting ASD in boys (Loomes et al., 2017). One potential explanation for this disparity is that much of the existing knowledge based on ASD has predominantly derived from research with autistic boys and men (Estrin et al., 2021; Watkins et al., 2014). In Canada, a recent report found that approximately 1 in 32 boys and 1 in 125 girls is diagnosed with ASD (Public Health Agency of Canada, 2022).

Due to recent advancements in our understanding of ASD, substantive changes were made to the *Diagnostic and Statistical Manual of Mental Disorders* (American Psychiatric Association, 2013). The diagnostic category Pervasive Developmental Disorder, which included Asperger’s Disorder, Childhood Disintegrative Disorder, Rett’s Disorder and Autistic Disorder, was removed and replaced with a single diagnosis of ASD. This change was made in light of the evidence that ASD is not a single condition but rather a spectrum of conditions characterized by differences in social skills, communication and behaviours. Scholars have argued, however, that other than acknowledging that girls without intellectual disability are less likely to receive an ASD diagnosis, the current *Diagnostic and Statistical Manual of Mental Disorders* (5th ed.; *DSM*-V) provides little guidance on how to address the under-diagnosis of females (Haney, 2016).

Currently, ASD refers to a range of neurodevelopmental disorders characterized by differences in social interaction and communication, and specific interests or repetitive behaviours (American Psychiatric Association, 2013). Increasingly, it is recognized that girls and women with ASD present differently than boys. For example, studies have shown that autistic girls are more likely to engage in reciprocal conversations, initiate friendships and have fewer social communication difficulties compared to boys (Beggiato et al., 2017; Hiller et al., 2014, 2016). There also appear to be gender differences in the presentation and frequency of repetitive and restricted behaviours. This research has found that girls tend to have fewer routines and limited restricted interests, and these behaviours also tend to be less frequent in girls compared to boys (May et al., 2016; Rubenstein et al., 2015; Szatmari et al., 2012). Since girls and women must often exhibit greater ASD characteristics and present with intellectual disability and mental health issues in order to receive a diagnosis, this creates challenges to the early detection of ASD (Tofani et al., 2022; Ward et al., 2022). The legacy of this situation may contribute to different educational outcomes for autistic boys and girls.

Research examining the impact of gender on educational outcomes in autistic students has been mixed. For example, while some studies have found that autistic girls have better executive functioning, academic achievement and school adjustment compared to boys (Bölte et al., 2011; de Giambattista et al., 2021; Serbin et al., 1990; Wong et al., 2015), other research has observed the opposite pattern (Frazier et al., 2014; Holtmann et al., 2007; Lemon et al., 2011; Sanchez et al., 2024). Studies examining post-secondary transition outcomes for autistic students have also yielded inconclusive findings. In a sample of 830 high school students in the United States, Chiang and colleagues (2012) did not find a significant link between gender and the likelihood of attending post-secondary. On the other hand, using population-based longitudinal data from Sweden, Stark et al. (2020) found that autistic girls were less likely to qualify for post-secondary than boys.

The aim of this descriptive study was to conduct secondary analyses on population-based, student-level administrative data collected in British Columbia (BC), Canada, to investigate the broad educational journeys of students who received an autism spectrum disorder (ASD) diagnosis and to explore gender differences in these journeys over time. Our study features the data’s novel capacity to follow students from Kindergarten (school entry) to Grade 12 or equivalent (school leaving), and onward onto post-secondary education. The specific objectives were to:

1. Investigate the time it takes for students to receive an initial ASD designation after starting school in Kindergarten;
2. Compute the frequency and percentage of students who stay longitudinally in the K-12 school system (are present in both Kindergarten and in Grade 12 or equivalent, years later) versus those who left the school system before reaching Grade 12 or equivalent;
3. Investigate the frequency and percentage of students who complete high school and, in turn, which school leaving credentials they earned; and
4. Compute the frequency and percentage of students who proceed to public post-secondary education (PSE) within BC, which PSE programme types they participate in, and their PSE transition time.

For analyses required for the third and fourth objectives, we retained only those students who stayed longitudinally in the K-12 system. In the last set of analyses, we retained only those students who further transitioned into PSE.

## Method

### Design

This study was a secondary analysis of population-based, student-level administrative data collected in British Columbia (BC), Canada. Data were collected by the BC Ministry of Education and Child Care (MECC), which oversees pre-Kindergarten and Kindergarten to Grade 12, and the BC Ministry of Post-Secondary Education and Future Skills (PSEFS), which oversees post-secondary education and includes various public post-secondary institutions that supplied their data to the Ministry of PSEFS. Data were pre-linked by the Ministries at the student level as part of their joint Student Transitions Project (STP) initiative. The STP project, which explores BC students’ high school graduates’ transitions into post-secondary education, is described by Heslop (2022) and by the Government of British Columbia (n.d.). The STP typically explores students’ transitions into public PS institutions within BC. The data do not capture information for students who attend public PS elsewhere or private PS institutions.

### Setting

The BC MECC regularly collects administrative-level data for all students attending government-funded schools. Students with disabilities and learning exceptionalities are assigned an inclusive education ‘designation’ or ‘category’ (British Columbia Ministry of Education and Child Care, 2016). This system is an administrative method to annually categorize and bookkeep individual K-12 students with their relevant support need(s) for the purpose of funding allotments to school districts.

One designation is autism spectrum disorder (ASD). In BC, there was a change of provincial policy on 1 January 2004 and of Ministry policy for the 2005/2006 school year. Beginning in 2005/2006, students received an ASD designation according to the updated Ministry guidelines (2016). Currently, the criteria for this designation include all of the following *Diagnostic and Statistical Manual of Mental Disorders* (4th ed.; *DSM* IV) and International Classification of Diseases, 10th Revision (ICD-10) categories: Autistic Disorder, PDD-NOS/Atypical Autism, Asperger Disorder/Syndrome, Rett Syndrome and Childhood Disintegrative Disorder. Before the policy change, it was simply referred to as ‘Autism’, not ‘Autism Spectrum Disorder’.

### Study population

The ASD study population included as the sample was obtained as part of a larger research initiative led by the first author to explore the educational journeys of BC’s students across assorted inclusive education designations. As part of this work, we received access to *N* = 68,684 anonymized and de-identified student records spanning 12 different designations, one of which was ASD. From the full data set, we retained the students flagged with ASD.

The ASD study population was divided into eight longitudinal cohorts, depending on when the student started Kindergarten. The final ASD study population, all cohorts combined, was *N* = 4282: 738 (17.2%) female and 3544 (82.8%) male (the Ministry does not collect information on students’ gender identity, only female/male gender). These 4282 students represent 6.2% of the *N* = 68,684 in the fuller set of all-designation inclusive education records. We did not have student age data, but most students in BC begin Kindergarten at age 5/6 years. Students also typically progress through grade levels at a rate of one grade per year (e.g. Kindergarten, Grade 1, Grade 2 and so forth). The count (%) of students with ASD by cohort is presented in Supplementary Table 7.

A given student may have received an ASD designation in any school year(s), not necessarily Kindergarten. Our study population was comprised of students whose *most common* designation over time, of the 12 possible designations, was ASD. Therefore, some students’ *most common* designation over time was ASD but may still have received one or more other designations through K-12, *not just ASD*.

Also, not all students receive a designation in every year while in the K-12 school system. Finally, in any given school year, only one designation may be assigned to a given student, typically that designation with the highest severity or need for support(s) (see British Columbia Ministry of Education and Child Care, 2016, for more information).

### Procedure

#### Duration of data

We were able to follow students up to and including the 2018/2019 school year (as it relates to their K-12 data). Hence, there are more years’ worth of longitudinal data available for students in the earlier cohorts versus the later ones. Students in later cohorts may have reached Grade 12 and/or completed school after 2018/2019, but we could not determine this definitively. This data limit presents a possibility of ‘right censoring’ (a data point is above a certain value but it is not known by how much) which may, in turn, influence certain outcomes. This censoring may particularly affect students in the later cohorts versus the earlier cohorts. Relatedly, some students who were not included in our analyses (because they had a most common designation that was not ASD) may have, eventually, been identified as having ASD and, in turn included in analyses, if the time duration of the data were longer than 2018/2019. But we would only be speculating as this information cannot be determined definitively. Finally, our data did not include students who may have in-migrated into the province’s K-12 school system after Kindergarten.

#### Primary measures

For time to initial ASD designation, we computed the difference between the school year in which a student received their initial ASD designation and the school year of their Kindergarten entry. The difference in these two values was the ‘wait or lag’ time to students’ initial ASD designation, yielding a continuous score. Note that, because of the diversity of designations the Ministry routinely tracks (including but not limited to ASD), there are many reasons why a given student may face a delay in receiving a designation. For the total ASD study population (*N* = 4282), *M* = 4.0, *SD* = 3.7, the range was 0 (received Ministry ASD designation in Kindergarten) to 14 years (received 14 years after Kindergarten). For females (*N* = 738), *M* = 4.5, *SD* = 4.0, the range was 0 to 14. For males (*N* = 3544), *M* = 3.9, *SD* = 3.6, the range was 0 to 14. We then grouped students’ differences into the following categories for the purpose of analyses: 0 year wait, 1–2 year wait, 3–4 year wait and 5+ year wait. A student could have received a different designation(s) before receiving their ASD designation. We report, however, only the time until the ASD designation, specifically.

For students who stayed longitudinally in K-12 (a student was present in the BC K-12 school system in Kindergarten at the start of their schooling and was also present in the system in Grade 12 or equivalent 12 years after Kindergarten) versus students who did not stay longitudinally (a student left the BC K-12 school system some time after Kindergarten, before completing K-12), we identified those who had both a Kindergarten flag and also a Grade 12 (or an equivalent) secondary ungraded flag in the school year in which students would be typically expected to have reached Grade 12 (i.e. 12 school years after Kindergarten). *Secondary ungraded* is when students take some number of Grade 8–12 level courses, but the school does not consider them to be in a specific grade level. We refer to a ‘flag’ as a column of data indicating which grade level a given student was in, in a given school year (e.g. Kindergarten).

Some students progressed through all K-12 grade levels one grade level per school year and reached Grade 12 or equivalent. Other individual student records did not stay in the system from K-12. In these cases, students may have dropped out of school, or moved out of (out-migrated from) the province prior to reaching or completing high school. The BC MECC database does not contain a variable explaining why certain students’ records cease before completion.

For students’ high school completion and credential earned, for those students who stayed longitudinally in the K-12 school system, we explored the rates of high school completion and also the attainment (or not) of specific high school leaving credentials students can earn in BC: *Adult Dogwood* (hereafter, BC Adult Graduation Diploma), *Evergreen* (BC School Completion Certificate) and *Dogwood* (BC Certificate of Graduation). Each of these graduation credentials have been described in the Supplementary File. We also explored those *missing* a school leaving credential altogether, despite staying longitudinally.

For students’ transitions to public post-secondary education in BC, we identified which students transitioned into Non-Developmental public post-secondary programmes versus Developmental programmes in British Columbia, for those students who stayed longitudinally. *Non-developmental* programmes refer to post-secondary programmes (e.g. Bachelor of Arts, Bachelor of Science, university transfer, Associates degree, criminology, engineering) for students who have met all provincial K-12 graduation requirements. *Developmental* programmes refer to post-secondary programmes (e.g. Adult Basic Education, employment training, food service careers, introduction to trades) for students who have met individual learning goals, but not necessarily all provincial K-12 graduation requirements.

We also computed students’ PSE transition times. Immediate means students transitioned within 0 or 1 calendar years of completing high school. Delayed means students transitioned 2 or more calendar years after. Other means any other timing (i.e. post-secondary started before K-12 completion).

#### Additional stratification measures

Assorted additional analyses involving not just students’ gender but also their cohort group and their number of designations (beyond ASD) were conducted to guide future studies as to how our primary results may change when assorted stratification effects are accounted or ‘controlled’ for. These analyses were carried out while working within a descriptive focus, considering the inherent limits of an administrative database with a restricted selection of variables, and accounting for a varying denominator size by measure.

We derived two dichotomous ‘stratification’ or ‘layering’ measures. This allows for three- (or-more) way tables, where categories of row and column variables are further subdivided by the stratification variable’s (variables’) own respective categories. This facilitates an exploration of how the relationship between row and column variables may alter when the stratification effects are accounted or ‘controlled’ for (IBM Corp, 2021). The two measures were as follows.

*‘Early cohort versus later cohort’* to explore potential cohort effects. We dichotomized students’ cohorts as follows: ‘Early cohort’ = 1 for students in the early cohorts 1/2/3/4 (1999/2000 to 2002/2003, respectively), and ‘early cohort = 0’ for students in the later cohorts 5/6/7/8 (2003/2004 to 2006/2007, respectively). With ‘early cohort’ *N* = 1843 (43.0%) and the ‘later cohort’ *n* = 2439 (57%), ASD students tended to be part of later cohorts versus earlier cohorts (supported by *n*s we provided by cohort). An additional two-way ‘early cohort/not’ × gender chi-square test was non-significant (χ^2^ = 0.39, *df* = 1, *p* = 0.532), indicating no association between the cohort group and gender. There was also a non-significant two-way chi-square of the original continuous/ungrouped cohort number and gender (χ^2^ = 2.89, *df* = 7, *p* = 0.895).

*‘Two or more designations versus one designation only’*. Students with two or more designations over time were coded ‘two or more’ = 1, and those with only one designation (ASD) were coded ‘two or more’ = 0. We created this variable to explore if there were potential effects of students being diagnosed with ‘only ASD’ versus multiple separate designations (including ASD) over time. Of the students with ‘one designation (ASD) only’, the sample size was *n* = 2442 (57.0%) and *n* = 1840 (43.0%) had ‘two or more designations, including ASD’. An additional two-way ‘2-or-more designations/not’ × gender chi-square test was non-significant (χ^2^ = 0.44, *df* = 1, *p* = 0.507), indicating no association between the number of designations group and gender.

#### Analytic plan

As detailed above, our study involved an exploration of assorted educational outcomes detailed in an administrative population-based database: (1) students’ time to initial ASD designation, (2) how many students stay longitudinally in the K-12 system versus not, (3) how many complete high school and, in turn, which school leaving credential they earn and (4) how many students proceed to public post-secondary within BC and, if so, which programmes they participate in, and their PSE transition time. Because each of these primary and additional stratification outcome measures had (or were derived on measures that had) varying scales of measurement (dichotomous, ordinal, continuous, nominal), we chose to use assorted descriptive statistical analyses, including frequencies and cross-tabulations. We also ran assorted two-way chi-square tests to examine statistically significant gender differences, one test for each primary measure of interest (thereby using *p* < 0.05 as our significance criterion in each).

Among the limited other information available in the administrative database, we used two derived stratification measures to produce assorted additional three- and four-way chi-square tables to explore if any gender differences in our primary measures potentially altered after layering. Because these additional analyses were not conducted as part of our primary objectives, but rather as additional exploratory analyses to inform future studies, we have reported in the tables that follow explicitly only the results of our primary analyses with descriptions of the additional analyses in the Results (with additional information provided in the Supplementary File). We have also reported, where relevant/possible, exact *p* values in instances where multiple testing may be necessary to further consider. All analyses were performed in SPSS Version 26 (IBM Corp, 2019).

### Permissions and ethics

We received written approval from the STP Secretariat (representing the BC MECC and the BC Ministry of PSEFS) and research ethics approval from the University of British Columbia Behavioural Research Ethics Board. All data were anonymized and de-identified. The data were only accessible within a Secure Research Environment (SRE) hosted by Population Data BC, a multi-university data and education resource at the University of British Columbia, and a data broker for the MECC and a data repository for the BC Ministry of PSEFS. There was no community involvement. We were required to mask all results with nine (9) or fewer cases to minimize risks of deductive disclosure.

## Results

### Objective 1: time to initial ASD designation after starting Kindergarten

Table 1 presents the frequency and percentage of all *n* = 4282 students with ASD for each of the assorted times to initial ASD designation we computed: 0 year wait (students received their ASD designation in Kindergarten), 1–2 year wait, 3–4 year wait and 5+ year wait after starting Kindergarten. It also presents the results by gender. A chi-square showed a significant gender difference in time to ASD designation (χ^2^ = 18.5, *df* = 3, *p* = 0.001). For both genders, nearly half of students waited 5 or more years to receive their ASD designation, with results suggesting that females were more likely to experience a longer delay than males.

**Table 1.** Students’ time to initial ASD designation after starting Kindergarten (*N* = 4282).

	Genders combined		Females only^a^		Males only^a^	
	*N*	%	*N*	%	*N*	%
0 year wait	1298	30.3%	223	30.2%	1075	30.3%
1–2 year wait	554	12.9%	77	10.4%	477	13.5%
3–4 year wait	581	13.6%	76	10.3%	505	14.2%
5+ year wait	1849	43.2%	362	49.1%	1487	42.0%

A gender × time to initial ASD designation chi-square was significant (χ^2^ = 18.5, *df* = 3, *p* = 0.001). See the Results Summary section for an explanation of this finding.

a Within-gender percentages.

An additional three-way time to initial ASD designation × gender × ‘early cohort/not’ chi-square (the last serving as the stratifier) indicated a partial association in which only students in the *later* cohorts had a significant time to initial ASD designation × gender association (χ^2^ = 18.4, *df* = 3, *p* < 0.001). A three-way time to initial ASD designation × gender × ‘2-or-more designations/not’ chi-square (the last serving as the stratifier) indicated a partial association in which students in *both* number of designations groups had a significant time to initial ASD designation × gender association (1 designation only: χ^2^ = 11.3, *df* = 3, *p* = 0.010; 2-or-more designations: χ^2^ = 10.4, *df* = 3, *p* = 0.016). A four-way time to initial ASD designation × gender × ‘early cohort/not’ × ‘2-or-more designations/not’ chi-square (the last two variables serving as the respective stratifiers) indicated a partial association in which there was a significant time to initial ASD designation × gender association for students in *both* number of designations groups, but *only* for those students in the *later* cohorts (1 designation only: χ^2^ = 10.3, *df* = 3, *p* = 0.016; 2-or-more designations: χ^2^ = 9.1, *df* = 3, *p* = 0.028). Because of this article’s focus on gender, we have placed additional results (if a Bonferroni correction was also applied given the stratification variables) in the Supplementary File for this objective and those that follow (where applicable).

### Objective 2: stayed longitudinally from K-12 versus not

Table 2 presents the frequency and percentage of all *n* = 4282 students with ASD by whether or not they stayed longitudinally in the K-12 school system. It also presents the results by gender. A total of 3802 (88.8%) students with ASD stayed longitudinally in K-12 over time. A chi-square showed no significant gender difference in staying longitudinally versus not (‘longitudinal/not’) (χ^2^ = 0.18, *df* = 1, *p* = 0.675).

**Table 2.** Students who stayed longitudinally from K-12 versus not (*N* = 4282).

	Genders combined		Females only^a^		Males only^a^	
	*N*	%	*N*	%	*N*	%
Not longitudinal	480	11.2%	86	11.7%	394	11.1%
Longitudinal	3802	88.8%	652	88.3%	3150	88.9%

A gender × longitudinal/not chi-square was not significant (χ^2^ = 0.18, *df* = 1, *p* = 0.675). The Results Summary section includes an explanation of this finding.

a Within-gender percentages.

Additional three- and four-way chi-squares (with longitudinal/not × gender) did not show a significant role of either the cohort group or the number of designations, either independently or when both variables were included simultaneously as stratifiers.

### Objective 3: high school completion and credential earned

Table 3 presents the frequency and percentage of all *n* = 3802 students with ASD who earned any completion credential [BC Adult Graduation Diploma, BC Certificate of Graduation and BC School Completion Certificate, combined] for students who had stayed longitudinally in the K-12 system. It also presents the results by gender. A chi-square showed a significant gender difference in completed high school (coded completed = 1) versus did not complete (coded completed = 0) (χ^2^ = 6.0, *df* = 1, *p* = 0.014). Male students with ASD were statistically more likely to complete high school (87.5% completed) than females (83.9% completed).

**Table 3.** Students’ high school completion: BC adult graduation diploma, BC certificate of graduation and BC school completion certificate, combined (*N* = 3802).

	Genders combined		Females only^a^		Males only^a^	
	*N*	%	*N*	%	*N*	%
Did not complete high school	500	13.2%	105	16.1%	395	12.5%
Completed high school (with any credential)	3302	86.8%	547	83.9%	2755	87.5%

A gender × completed/not chi-square was significant (χ^2^ = 6.0, *df* = 1, *p* = 0.014). See the Results Summary section for an explanation of this finding.

a Within-gender percentages.

An additional three-way ‘completed/not’ × gender × ‘early cohort/not’ chi-square indicated a partial association in which only students in the *later* cohorts had a significant ‘completed/not’ × gender association (χ^2^ = 4.2, *df* = 1, *p* = 0.041). A three-way time to ‘completed/not’ × gender × ‘2-or-more designations/not’ chi-square indicated a partial association in which only students with *one designation* only had a significant ‘completed/not’ × gender association (χ^2^ = 4.1, *df* = 1, *p* = 0.042). A four-way time to ‘completed/not’ × gender × ‘early cohort/not’ × ‘2-or-more designations/not’ chi-square did not show a significant role of either the cohort group or the number of designations group.

Table 4 presents the frequency and percentage of all *n* = 3802 students with ASD who earned each of the *specific* school completion credentials in BC, for students who had stayed longitudinally in the K-12 system. It also presents the results by gender. We also included students who were *missing* a school leaving credential, despite being in the K-12 school system longitudinally. A chi-square showed a significant gender difference in the specific high school credential earned (χ^2^ = 16.0, *df* = 3, *p* = 0.001). Male students with ASD were statistically more likely to complete high school with a BC Certificate of Graduation credential (56.5% received a BC Certificate of Graduation) than females (48.3%). The BC Certificate of Graduation is the credential most associated with post-secondary attendance (Heslop, 2022).

**Table 4.** Students’ specific high school credential earned (*N* = 3802).

	Genders combined		Females only^a^		Males only^a^	
	*N*	%	*N*	%	*N*	%
Missing credential	500	13.2%	105	16.1%	395	12.5%
BC Adult Graduation Diploma	220	5.8%	39	6.0%	181	5.7%
BC School Completion Certificate	986	25.9%	193	29.6%	793	25.2%
BC Certificate of Graduation	2096	55.1%	315	48.3%	1781	56.5%

A gender × credential type chi-square was significant (χ^2^ = 16.0, *df* = 3, *p* = 0.001). The Results Summary section includes an explanation of this finding.

a Within-gender percentages.

An additional three-way credential × gender × ‘early cohort/not’ chi-square indicated a partial association in which only students in the *early* cohorts had a significant credential × gender association (χ^2^ = 15.3, *df* = 3, *p* = 0.002). A three-way credential × gender × ‘2-or-more designations/not’ chi-square indicated that only students with *two or more designations* had a significant credential × gender association (χ^2^ = 13.1, *df* = 3, *p* = 0.004). A four-way credential × gender × ‘early cohort/not’ × ‘2-or-more designations/not’ chi-square indicated that there was a significant credential × gender association only for students who had two or more designations *and* who were also in an early cohort (χ^2^ = 14.9, *df* = 3, *p* = 0.002).

### Objective 4: transitions to public post-secondary education in BC: transitioned versus did not transition, programme and transition timing

Table 5 presents the frequency and percentage of all *n* = 1704 students with ASD who transitioned into either a non-developmental or developmental public post-secondary programme in British Columbia, for students who had stayed longitudinally in the K-12 system. It also presents the results by gender. A ‘transitioned/not’ (coded transitioned = 1, did not transition = 0) × gender chi-square showed no significant difference (χ^2^ = 1.31, *df* = 1, *p* = 0.253).

**Table 5.** Students’ transitions to public post-secondary education in BC (*N* = 3802).

	Genders combined		Females only^a^		Males only^a^	
	*N*	%	*N*	%	*N*	%
Did not transition to public post-secondary education in BC	2098	55.2%	373	57.2%	1725	54.8%
Transitioned to public post-secondary education in BC	1704	44.8%	279	42.8%	1425	45.2%

A gender × transitioned/not chi-square was not significant (χ^2^ = 1.31, *df* = 1, *p* = 0.253). See the Results Summary section for an explanation of this finding. Transitioned into either a non-developmental or developmental PSE programme at a BC public post-secondary education institution.

a Within-gender percentages.

Additional three- and four-way chi-squares (with transitioned/not × gender) did not show a significant role of either the cohort group or the number of designations, either independently or when both variables were included simultaneously as stratifiers.

Table 6 presents the frequency and percentage of all *n* = 1704 longitudinal students with ASD who transitioned into either a non-developmental (coded programme = 1) or developmental (coded programme = 0) public post-secondary programme in British Columbia. It also presents the results by gender. A chi-square showed no significant gender difference in transitioned into a non-developmental programme versus a developmental programme (χ^2^ = 0.67, *df* = 1, *p* = 0.414).

**Table 6.** Students’ transition time to public post-secondary education in BC (*N* = 1704).

	Genders combined		Females only^a^		Males only^a^	
	*N*	%	*N*	%	*N*	%
Non-developmental	1267	74.4%	202	15.9%	1065	84.1%
Delayed	258	20.4%	37	18.3%	221	20.8%
Immediate	988	78.0%	159	78.7%	829	77.8%
Other	21	1.7%	*Masked*	*Masked*	15	1.4%
Developmental	437	25.6%	77	17.6%	360	82.4%
Delayed	122	27.9%	26	33.8%	96	26.7%
Immediate	311	71.2%	49	63.6%	262	72.8%
Other	*Masked*	0.9%	*Masked*	*Masked*	*Masked*	*Masked*

Chi-square showed no significant gender difference in transitioned into non-developmental versus developmental programme (χ^2^ = 0.67, *df* = 1, *p* = 0.414). The Results Summary section includes an explanation of this finding. *Non-developmental*: Chi-square showed no significant gender difference in transition time (χ^2^ = 3.01, *df* = 2, *p* = 0.222). *Developmental*: Chi-square showed no significant gender difference in transition time (χ^2^ = 4.78, *df* = 2, *p* = 0.092). See the Results Summary section for an explanation of this finding.

a Within-gender percentages for transition time. Other percentages within programme type (non-developmental vs developmental).

An additional three-way programme × gender × ‘early cohort/not’ chi-square did not indicate any significant role of cohort group. A three-way programme × gender × ‘2-or-more designations/not’ chi-square indicated that only students with *two or more designations* had a significant programme × gender association (χ^2^ = 7.8, *df* = 1, *p* = 0.005). A four-way programme × gender × ‘early cohort/not’ × ‘2-or-more designations/not’ chi-square indicated that there was a significant programme × gender association only for students with two or more designations *and* who were also in an early cohort (χ^2^ = 6.6, *df* = 1, *p* = 0.010).

Table 6 also presents the timing of students’ transition (immediate, delayed, other) by programme type and gender. Separate chi-squares performed for non-developmental and developmental indicated no significant gender difference in transition time by PSE programme type (non-developmental: χ^2^ = 3.01, *df* = 2, *p* = 0.222; developmental: χ^2^ = 4.78, *df* = 2, *p* = 0.092).

Regarding the *n* = 1267 *non-developmental* programme transitioners, an additional three-way timing × gender × ‘early cohort/not’ chi-square did not indicate any significant role of cohort group. A three-way timing × gender × ‘2-or-more designations/not’ chi-square did not indicate any significant role of the number oSf designations group. A four-way timing × gender × ‘early cohort/not’ × ‘2-or-more designations/not’ chi-square indicated that there was a significant timing × gender association only for students with two or more designations *and* who were also in a *later* cohort (χ^2^ = 6.8, *df* = 2, *p* = 0.034).

Regarding the *n* = 437 *developmental* programme transitioners, additional three- and four-way chi-squares did not show a significant role of either the cohort group or the number of designations, either independently or when both variables were included simultaneously as stratifiers.

#### Results summary

With genders combined, results indicate that 4282 students began Kindergarten whose most common designation *over the course of their K-12 schooling* was ASD. Of these 4282 students, 3802 (88.8% of 4282) stayed longitudinally in the K-12 school system over time.

Of these 3802 students, 3302 (86.8% of 3802) (or 77.1% of the original 4282 students) earned a high school leaving credential, while 500 (13.2%) (or 11.7% of the original 4282 students) were missing a credential despite being longitudinally in the K-12 school system. Also of these 3802 students, 1704 (44.8% of 3802) (or 39.8% of the original 4282 students) transitioned into either a non-developmental or a developmental public post-secondary programme in BC, with varying transition times, while 2098 (55.2%) (or 50.0% of the original 4282 students) did not transition to PSE at all.

Of the 1704 PSE transitioners, 1267 (74.4%) entered a non-developmental PSE programme (29.6% of the original 4282 students), while 437 (25.6%) entered a developmental PSE programme (10.2% of the original 4282 students).

Regarding our *primary* measures of interest, we found that females were more likely than males to experience a longer delay in obtaining an initial ASD designation. We found that there were no significant differences whether or not students stayed longitudinally from Kindergarten to Grade 12. Furthermore, we found that males were more likely to complete high school than females and were more likely to complete high school with a BC Certificate of Graduation credential than females. With respect to whether or not student transitioned to post-secondary and whether or not student transitioned to a non-developmental versus a developmental post-secondary programme, there were no significant gender differences between males and females. Finally, regarding the timing of students’ transitions to post-secondary, there were no significant gender differences.

Regarding our additional *stratification* analyses, we found emerging evidence showing that the role of early/later cohort, and also the number of designations a student had, altered some associations across the primary measures. In the case of whether a student stays longitudinally/not, and also transitioned to post-secondary/not, there was no significant role of cohort group and number of designations group. There was also no significant role of these stratifiers for the *n* = 437 *developmental* programme transitioners’ post-secondary transition timing. There were, however, either partial or full associations found for students’ time to initial ASD designation, whether/not they completed high school, the specific high school leaving credential earned, the programme type of post-secondary programme transitioners (non-developmental and developmental) and the 1267 *non-developmental* programme transitioners’ post-secondary transition timing. These findings, however, must be considered in the context that some of these significant effects no longer remain if a Bonferroni correction is applied (see Supplementary File). Therefore, please interpret additional results with caution.

## Discussion

Consistent with previous research in the United States (Giarelli et al., 2010; Maenner et al., 2020; Shattuck et al., 2009; Windham et al., 2011), female students were more likely to experience delays in obtaining an ASD designation. It is possible that gender stereotypes influence perceptions of ASD characteristics, contributing to delays in diagnosing females (Geelhand et al., 2019). Cultural norms and societal expectations can shape how males and females are expected to behave, which can create challenges identifying ASD in females (Kreiser & White, 2014). Alternatively, others have suggested that because the bulk of the existing research has comprised mostly male samples (Estrin et al., 2021; Watkins et al., 2014), there is a lack of understanding among professionals about how ASD presents in females (Bargiela et al., 2016). For example, girls and women more frequently ‘camouflage’ their behaviours (Hull et al., 2017) in order to ‘fit in’ to social situations, making it more difficult to detect ASD characteristics. This camouflaging has been shown to take significant cognitive and emotional effort, negatively impacting their psychological and emotional well-being (Beck et al., 2020). Our results highlight the critical need for improved diagnostic tools, early identification and tailored supports which address females’ unique experiences and facilitate access to services (Bent et al., 2020). Given the role of school staff in recognizing ASD, the results emphasize the need for updated education on gender differences in ASD presentation.

The present study also found that male students with ASD were statistically more likely to complete high school (87.5% completed) than females (83.9% completed). Male students with ASD were also more likely to complete high school with a BC Certificate of Graduation credential (56.5%) than females (48.3%). These findings indicate that although current high school graduation rates for students with ASD appear to be increasing (Liptak et al., 2011; Wei et al., 2014), the gender disparity particularly disadvantaging female students is concerning and demands further attention. While ASD is not the sole factor contributing to inequities, our findings support the need for targeted interventions and policies to achieve equitable educational outcomes for female students with ASD, which is further emphasized when considering that educational attainment is a social determinant of health and well-being (Raphael, 2016).

There were no significant gender differences found in whether students transitioned into post-secondary or not (non-developmental or developmental programme). This suggests that some of the disadvantages that female students with ASD experience in high school diminish if they are able to transition to post-secondary. A possible explanation for this finding is that female students with ASD who are transitioning to post-secondary may be students who required fewer supports in grade school. It is also plausible that the majority of these female students transitioning to post-secondary graduated with the BC Certificate of Graduation credential and as a result have greater adaptive skills and propensity to overcome challenges and barriers contributing to gender disparities. Indeed, previous research, including a study by Ji et al. (2023), observed fewer gender differences in higher IQ students, particularly in the domains of perceptual reasoning and reciprocal social interaction, compared to lower IQ students.

### Limitations

There are a number of limitations that warrant caution when interpreting the findings of this study. We are not able to draw conclusions from the data regarding the reasons for students discontinuing education before high school completion. It is possible that these students graduated from Grade 12 beyond the 2018–2019 endpoint and are thus not captured in the current dataset. The data to which we had access do not include students who pursued post-secondary education at private institutions within BC or post-secondary education outside of BC and consequently their transition to post-secondary information is unknown. Available data, however, suggest that students in BC tend to remain within the province (Heslop, 2010), a pattern likely similar for students with ASD. We were also restricted by the demographic data collected by the data owner, a common issue when analysing administrative databases (Connelly et al., 2016), and therefore many variables that may influence educational outcomes (e.g. socio-economic status, race, additional medical diagnoses) were not part of our analysis.

Our study highlights the value of longitudinal, population-based and student-level data in conducting gender-based analyses in ASD research. Examining gender differences in educational outcomes longitudinally is crucial as the prevalence of students with ASD in mainstream education programmes continues to grow in Canada (Public Health Agency of Canada, 2022). Future research directions should focus on data linkage, which would allow researchers to include a broader array of important variables when considering the educational journeys of these students. Besides, additional analytic approaches, such as time-to-event analyses, could provide further insights into students’ journeys.

In conclusion, understanding the influence of gender on the academic journeys of students with ASD is a growing area of inquiry. Our study contributes to this area of scholarship and, along with other empirical studies, reinforces the need for early screening and access to assessments, as well as the development of tailored interventions and services. Such research is needed to promote more equitable educational experiences and outcomes for all.

## Supplemental Material

sj-docx-1-aut-10.1177_13623613251345532 – Supplemental material for Population-level gender-based analysis of the educational journeys of students with autism spectrum disorder in British Columbia, Canada Supplemental material, sj-docx-1-aut-10.1177_13623613251345532 for Population-level gender-based analysis of the educational journeys of students with autism spectrum disorder in British Columbia, Canada by Jennifer Baumbusch, Jennifer EV Lloyd and Vanessa C Fong in Autism
