# Supplementary material for: Population-level gender-based analysis of the educational journeys of students with autism spectrum disorder in British Columbia, Canada
Source: Autism. 2025 Jun 21;29(10):2550–61. doi: 10.1177/13623613251345532 (PMC12417614; doi:10.1177/13623613251345532)
Supplement: sj-docx-1-aut-10.1177_13623613251345532 – Supplemental material for Population-level gender-based analysis of the educational journeys of students with autism spectrum disorder in British Columbia, Canada [file sj-docx-1-aut-10.1177_13623613251345532.docx]

**Supplementary Material**

*Table 7: Frequency and Percentage of Students with ASD by Cohort (N = 4,282)*

| *Cohort* | *N_Total_* | *N_Females_* | *%_Females_* | *N_Males_* | *%_Males_* |
| --- | --- | --- | --- | --- | --- |
| *Cohort 1: K in 1999/2000* | 433 | 79 | 18.2% | 354 | 81.8% |
| *Cohort 2: K in 2000/2001* | 423 | 67 | 15.8% | 356 | 84.2% |
| *Cohort 3: K in 2001/2002* | 486 | 83 | 17.1% | 403 | 82.9% |
| *Cohort 4: K in 2002/2003* | 501 | 81 | 16.2% | 420 | 83.8% |
| *Cohort 5: K in 2003/2004* | 546 | 95 | 17.4% | 451 | 82.6% |
| *Cohort 6: K in 2004/2005* | 551 | 101 | 18.3% | 450 | 81.7% |
| *Cohort 7: K in 2005/2006* | 667 | 108 | 16.2% | 559 | 83.8% |
| *Cohort 8: K in 2006/2007* | 675 | 124 | 18.4% | 551 | 81.6% |
| *All Cohorts Combined* | 4282 | 738 | 17.2% | 3544 | 82.8% |

*Note 1.* K = Kindergarten

*Note 2.* Percentages presented within-cohort.

*British Columbia School Leaving Credentials*

*Adult Dogwood* (BC Adult Graduation Diploma) is typically for students 19 years and older who completed all provincial graduation requirements.

*Evergreen* (BC School Completion Certificate) is typically for students with moderate/profound intellectual disability who met individual learning goals, and is therefore not a graduation credential.

*Dogwood* (BC Certificate of Graduation) is typically for students 18 years and younger who completed all provincial graduation requirements -- also the credential most associated with post-secondary attendance (Heslop, 2022).

*Bonferroni Corrections*

Because of this paper’s focus on gender as a primary measure, we have placed results -- if a Bonferroni correction were also applied -- here, where applicable:

**Objective 1: Time to Initial ASD Designation after Starting Kindergarten**

Given that there are three additional tests for each primary measure, the significance criterion would change to *p* = .017, and all aforementioned stratification chi values would remain significant with the exception of the last.

**Objective 3: High School Completion and Credential Earned**

First set of analyses: If Bonferroni-corrected, neither stratification chi value would remain significant.

Second set of analyses: If Bonferroni-corrected, all aforementioned stratification chi values would remain significant.

**Objective 4: Transitions to Public Post-Secondary Education in BC: Transitioned versus Did not Transition, Program, and Transition Timing**

First set of analyses: If Bonferroni-corrected, all aforementioned stratification chi values would remain significant.

Second set of analyses: If a Bonferroni-corrected significance criterion were changed to *p* = .017, the one aforementioned significant stratification chi value would no longer remain significant.
